# Threatening and nurturing mental health: insights from Danish elite athletes on the dynamic interplay of factors associated with their mental health

**DOI:** 10.3389/fspor.2024.1451617

**Published:** 2024-11-27

**Authors:** Andreas Küttel, Louise K. Storm, Natalia Stambulova, Kristoffer Henriksen

**Affiliations:** ^1^ Department of Sport Sciences and Clinical Biomechanics, University of Southern Denmark, Odense, Denmark; ^2^ School of Health and Welfare, Halmstad University, Halmstad, Sweden

**Keywords:** mental health, elite sport, qualitative, protective and risk factors, environment

## Abstract

**Introduction:**

Numerous factors have been identified that potentially influence athletes' mental health. Given the predominant focus in the literature on athletes' mental health risk factors, our study aimed to explore elite athletes' perceptions of factors associated with their mental health and thriving based on the combination of holistic developmental and ecological approaches.

**Methods:**

Seven Danish international elite athletes representing diverse sports were interviewed twice. The initial interview delved into their retrospective perspectives on career and mental health development, while the subsequent interview, conducted two months later, centered on recent events.

**Results:**

Thematic analysis yielded a map outlining four overarching themes. Elite sport was perceived as a (1) *relentless performance context* marked by rigorous demands, which evoked (2) *personal reactions* among athletes characterized by heightened expectations, self-blame, and anxiety. In response to these challenging demands, athletes have cultivated (3) *coping resources and strategies* over the course of their careers, such as self-reflection, emphasis on recovery, planning and prioritization skills, and passion for their sport. Nonetheless, the development of these resources and strategies was a gradual process, often informed by past experiences of mental health difficulties during adolescence. Additionally, they have found support for their mental health within a (4) *nurturing environment* consisting of supportive coaching, camaraderie among teammates, guidance from experts, and caring relationships.

**Discussion:**

The findings of this study highlight the complex interplay of factors affecting mental health and emphasize the need for creating supportive environments that help athletes manage the intense demands of elite sport.

## 1 Introduction

Elite athletes are often celebrated for their exceptional physical abilities and achievements in the world of sports. However, concerns have publicly been raised over the high prevalence of mental health disorders among elite athletes due to the demanding nature of elite sports, including intense training and competitions, injuries, deselection, and public scrutiny (1, 2). In prevalence studies, researchers typically utilized cross-sectional designs to elucidate the incidence rates of mental health disorders (e.g., depression, anxiety, and eating disorders) in correlation with specified risk factors (for reviews, see (3, 4). However, this approach provides a limited understanding of factors that help athletes to protect and nurture mental health and neglects the dynamic nature of mental health (1).

Mental health is a broad concept and is sometimes used to describe a desirable mental condition of well-being, and at other times it is used to describe mental ill-being or mental disorders (1). In this study, we refer to the following definition that has been proposed by Kuettel and Larsen (3) in relation to elite sport:

Mental health is a dynamic state of well-being in which athletes can realize their potential, see a purpose and meaning in sport and life, experience trusting personal relationships, cope with common life stressors and the specific stressors in elite sport, and are able to act autonomously according to their values (p. 23).

In their scoping review of research on mental health risk and protective factors in elite athletes, Kuettel and Larsen (3) identified 82 factors and grouped them into four categories creating a framework to be used in this study. These categories are: (a) personal risk factors (e.g., adverse life events, injury and overtraining, and low social support); (b) personal protective factors (e.g., protective behaviors, positive social relationships, and recovery); (c) sport-environmental risk factors (e.g., sport-specific stressors, stigma towards help-seeking); and (d) sport-environmental protective factors (e.g., mental health literacy and support, trusting sport climate). While only 11% of the 43 empirical studies in this review were qualitative, these studies contributed to over half of the factors perceived by athletes as associated with their mental health. Another review looked at the mental health of student-athletes (5) and divided the many factors identified in (a) demographic, (b) generic (e.g., sleep quality, stress, social support); (c) sport-specific; and (d) dual career specific factors. Both reviews conclude that the factors affecting athletes’ mental health are numerous and originate from various personal development and environmental levels, pointing to the complexity of the issue. The authors requested further qualitative studies to provide insights into the complexities of athletes' lived experiences and the mental health factors interplay. The present study was designed with this quest in mind.

Within the athlete career sport psychology discourse (6), elite athletes' mental health is considered based on the holistic developmental and ecological approaches, that is as incorporated into their multilevel development and accommodation with related micro-and macro-environments. This integrated approach acknowledges mental health as both a resource and an outcome of career development, leading to the conceptualization of career excellence as “an athlete's ability to sustain a healthy, successful, and long-lasting career in sport and life” (6, p. 14). Throughout their careers, athletes proceed through different sporting environments – each with its unique culture – that can either nourish or malnourish their mental health (7). Stigmatization within elite sports culture can discourage individuals from seeking help as sports organizations might view mental health issues as an undesirable weakness inconsistent with high-level sports (8). Contrary, mental health literacy (9), encompassing the knowledge, attitudes, and skills needed to promote mental well-being and complementing support, can play a pivotal role in recognizing and addressing mental health difficulties both among athletes and within their support networks (10).

In Denmark, elite sport guided by the current law (11) should be conducted in a socially responsible manner supporting the welfare and education of elite athletes. There has historically been a focus on athletes' dual career (i.e., combining sports with study/work) and creating supportive talent development environments in Denmark with an emphasis on mental health (e.g., 12, 13). However, scandals within elite swimming have exposed unethical practices such as public weighing and bullying, leading to psychological distress in several athletes. This has sparked a public and political debate about the role of mental health in elite sport and led to the development of the Team Denmark applied model of mental health (14). The model underscores the importance of extending focus beyond individual athletes and their daily routines, urging consideration of the training environment and leadership in mental health prevention, early detection, and referral. In a recent quantitative study among Danish elite athletes, three distinctive mental health profiles (i.e., flourishing, moderate mental health, and languishing) were discovered (15). Most athletes were flourishing, and differences in perception of stressors, social support, and the role of their sporting environment were evident between athletes in the different profiles. Yet, these findings provided only a snapshot of the mental health state of athletes and certain contributing factors. Therefore, to deepen our understanding of contributing mental health factors in a holistic and developmental perspective, this study was guided by the following research question: *How do Danish elite athletes perceive the dynamic interplay of personal and environmental factors as influencing their mental health, specifically in terms of risk (negative/threatening) and protective (positive/nurturing) factors, within the context of their mental health and career development?*

## 2 Material and methods

### 2.1 Philosophical underpinning

We grounded our research in ontological relativism which posits that reality is multifaceted, created, and dependent on the mind. Additionally, we adopted a socio-constructionist paradigm, recognizing that knowledge is shaped by cultural influences and relational interactions, rather than being objectively observed or discovered (16). This perspective entails that we as researchers always bring our personal background and experience into the knowledge construction (17).

### 2.2 Researchers, participants, and data collection

To increase the credibility of our claims, we share the research group experiences. The first author is a former elite athlete who competed in three Olympic Games. Upon athletic retirement he completed a PhD focusing on athletes' careers and transitions. He also provides sport psychology consulting to elite athletes and teams. The co-authors are all scientist-practitioners in youth and elite sports, as well as experienced qualitative researchers in sport and exercise psychology.

#### 2.2.1 Participants

We aimed for information-rich cases of elite athletes who were willing to share personal insights in their careers and mental health. Potential participants were recruited through Team Danmark's sport psychologists and included athletes that had experienced difficulties with their mental health in the past. The sample consisted of seven Danish elite athletes (including four females), who competed at the pinnacle of international competitions, such as the Olympics, World Championships, or equivalent levels in professional sports (18). These athletes represented a diverse range of sports (athletics, badminton, kayaking, cycling, handball, soccer). The athletes were on average 26,7 years of age (range: 23–38 years) and all had experience with a dual career. Two athletes had recently turned professional, while the other five were balancing their elite sports careers with university studies. This sample was deemed to provide sufficient information power (19) to address the study aim.

#### 2.2.2 Procedure

After receiving approval from the regional ethics committee, potential athletes were contacted via sport psychologists of Team Denmark (Danish elite sport governing body). Participants were informed about the scope of the study and ethics with written consent provided before the first interview. Each athlete was interviewed twice. The first interview was conducted face-to-face and took on average 85 min (range 65–107 min). The follow-up interview was conducted online due to athletes travel commitments via Zoom roughly two months after the first interview and lasted on average 40 min. The first interview took a retrospective approach, focusing on past events and the associated risk and protective factors for mental health. In contrast, the second interview centred on more recent events, and allowed for a deeper exploration of specific topics mentioned in the first interview (e.g., relationship with coach, strategies for recharging, coping with injury).

#### 2.2.3 Interviews

For the first interview, a semi-structured interview guide was developed that contained five parts. The *first part* served as an icebreaker. The interviewer shortly described his own sporting background and asked about the interviewee's career and present situation, for example: “Can you tell me a little about your sporting and family background? What is happening in your life right now?”. The *second part*, focused on their understanding of mental health: “When you hear mental health, what pops up in your mind?”. In the *third part*, we discussed different mental health definitions to establish a common understanding before continuing with the interview. The *fourth part* dealt with the specific context of elite sport in relation to mental health: “How is mental health a topic in your team or your collaboration with coaches?”. In the *fifth part*, applying a biographical mapping method (20), athletes were asked to draw a line of their perceived development of mental health level as well as their subjective performance level using different colours and mark significant life events that they thought were influential for either their mental health or performance: “Can you tell me more about the particular event/experience you marked here? How did it influence your mental health?”. The participants' line drawings and annotations were subsequently used as reference points in the interview to stimulate responses and to invite the athletes to make sense of the dynamics of mental health and related incidents. In the second interview, we explored recent mental health fluctuations in connection with current events. With the rapport and trust established in the first interview, we were able to delve more deeply into sensitive topics previously mentioned (“Let's talk again about this period when you were injured and had a lot of self-doubts…”), adding an additional layer of reflection on the protective and risk factors.

### 2.3 Data analysis, representation, and reflections on rigor

During the reflexive thematic analysis, we were guided by the six steps (i.e., familiarizing with data, initial coding, searching for themes, reviewing themes, defining and naming themes, and producing the report) suggested by Braun and Clarke (19). Given that the first author led on every aspect of the data collection process, he was immersed in the data prior to formal analysis reading and listening to the recordings and making preliminary notes in the reflexive journal (Step 1). The preliminary iteration of coding (Step 2) was conducted in NVivo14 for each of the interviews by identifying data that could be useful in answering the research questions. Throughout this process, the first author double-coded each interview in accordance with the semantic meaning communicated by participants, and the latent meaning that was interpreted by writing a short summary (21). This was guided by a predominantly inductive approach to best represent meanings communicated by the athletes. However, a deductive analysis was performed to ensure that the inductive coding was relevant to the study's aim and its underpinning four categories framework (3).

Once all the data were coded, in Step 3, the focus shifted from the interpretation of individual data to aggregating meaningful ideas across the dataset. These initial codes were subsequently grouped around themes as patterns of shared meaning that were internally coherent, consistent, and distinctive (19). In Step 4, themes were constructed as creative and interpretive stories about data (16) in a process going back-and forth between the data and the theoretical framework (3). As Braun and Clarke (19) emphasize, themes do not passively emerge from the data, and neither are they waiting to be simply identified. Rather, themes are creative and interpretive stories about the data and produced at the intersection of the researcher's theoretical assumptions, their analytic resources and skill, and the data themselves. To ensure the analytical rigor, we drew upon the key indicators including topic worthiness, member reflections, significant research contribution, naturalistic and transferable generalizability, critical friends, and reflexivity (22). During the analytical process (Step 5), co-authors and former elite athletes now working in academia acted as critical friends who encouraged the first author to reflect upon the interpretation of the data, the thematic map, as well as exploring alternative explanations. Last, we re-worded the themes in a collaborative effort to represent them as the athletes’ voices to be presented in the text and the thematic map (Step 6).

## 3 Results

**Figure 1:**
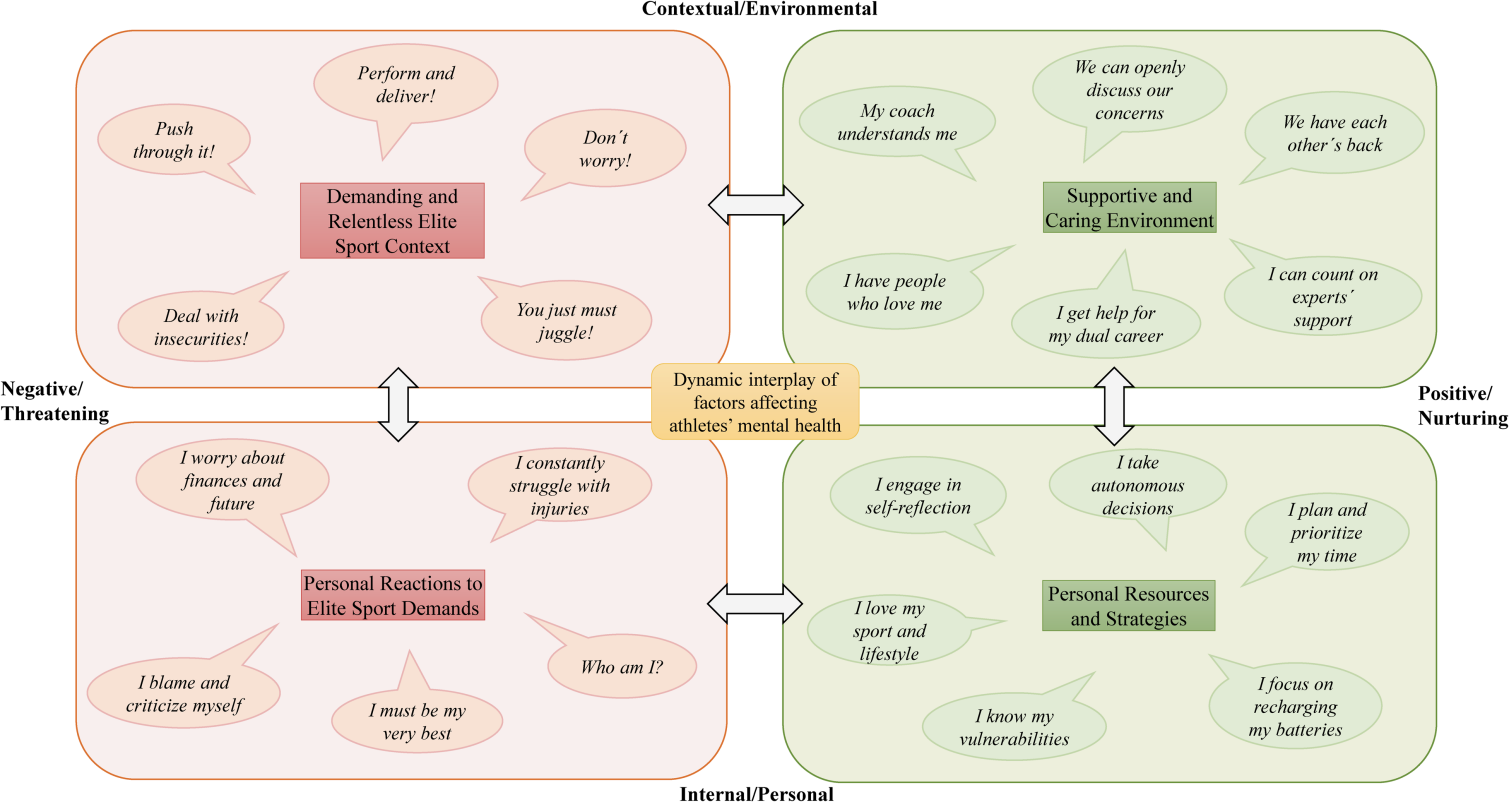
Qualitative map of factors Danish elite athletes associate with their mental health.

The findings are structured around four overarching themes, comprising a total of 22 sub-themes that were developed from the interviews. The four main themes are (a) demanding elite sport context, (b) personal reactions to elite sport demands, (c) personal resources and strategies, and (d) supportive and caring environment. We refer to the sub-themes as factors portraying athletes' positive or negative associations with their mental health. These are visually represented in Figure 1 as a thematic map.

### 3.1 Demanding and relentless elite sport context

Within this theme, the elite sport context and its culture were sometimes perceived as a relentless performance context where athletes felt they must (a) push through no matter what, (b) perform and deliver on a daily basis, (c) tolerate not to being taken seriously, (d) juggle with all kinds of different demands and expectations, and (e) deal with the insecurities inherent in elite sport.

**Push Through It!** Through acculturation in the elite sport context along their careers, athletes learned that within elite sport, one is expected to have a tough mindset, show no weakness, pushing through injuries, and putting in relentless effort. Participant 2 (P2) shared the advice he received when dealing with serious issues in his private life: “..well, you just have to pedal it away!”. P7 perceived his old team “as a macho-culture where you don't talk about difficult things.” Mental health problems were then not taken seriously, and athletes felt that they were expected to wear a mask and deal with problems on their own.

**Don't Worry!** During periods when athletes were struggling and tried to articulate their mental health difficulties, they perceived a lack of serious consideration and felt that their concerns were merely brushed aside, particularly evident within coach-athlete relationships where trust and closeness were lacking. P1 asked herself: “Why do we avoid discussing the things that don't work out? And then you get a superficial response like: Don't worry, everything will be fine…”. Athletes believed that it is not solely their responsibility to discuss concerns, rather, coaches should also possess the ability to recognize issues and proactively address them in a timely manner, demonstrating authentic interest and engagement in the athlete. Athletes emphasized that when their coaches provided superficial responses, it can lead them to overlook their own mental health issues.

**You Just Must Juggle!** Athletes felt pressured from the demands and expectations stemming from the various life domains, especially in relation to the dual career in their late teenage years that also included a lot of commuting between home, school, and training. P6 vividly remembered this period: “I was expected to be in four different places at the same time. I had no energy left and was rarely at home; it was incredibly stressful”. Looking back, all athletes remembered this phase as a chaotic period in their lives where their mental health was under pressure, and they felt like they were merely surviving rather than thriving. The athletes found it challenging to set boundaries and to recognize when they had reached their limits which caused frustration and mental breakdowns. They expressed a sense of being left to fend for themselves, as no one seemed to have a comprehensive understanding of their overall situation.

**Deal With Insecurities!** Athletes discussed various external factors associated with their athletic career that are beyond their control and were perceived as detrimental to their mental health. P4 explains: “At the end of the last season, my coach announced he stops. This sparked a multitude of uncertainties about the future, leading me to question whether I should quit, continue, or if my last race had already been history…”. Athletes often felt helpless and left alone to deal with the uncertainties and insecurities inherent in elite sport, such as lack of transparency about selection procedures, unexpected changes in coaching, and the risk of contract termination.

### 3.2 Personal reactions to elite sport demands

This theme relates to the struggles that athletes experience with their involvement in elite sport and includes factors they perceive as negatively affecting their mental health. These internal factors include (a) worries about finances and future, (b) the constant struggle with injuries, (c), identity issues, (d), high personal standards, and (e) self-blame and criticism.

**I Worry About Finances and Future.** Despite being among the top in Danish elite sport, most athletes find it difficult to make a living from their sport, and some still rely on financial support from their parents or partner. Athletes expressed insecurity and financial concerns, especially if their income depends on prize money, which is never guaranteed and cannot be earned during periods of injury. Athletes conveyed existential fears when facing uncertainty related to contract renewal, as they are responsible for supporting their families: “My contract expires in just four weeks, and I’m getting more and more concerned. With a wife and two kids to support, I find myself becoming more easily irritable, and I’m filled with anxiety about what lies ahead.” (P7).

**I Constantly Struggle With Injuries.** Every interviewed athlete talked about episodes when they were bothered by either acute or overuse injuries, or general physical health problems such as a virus infection that put them out of the game for a long time. Even though athletes were aware that it is quite normal to be injured, they have difficulties acknowledging it. “There's an expectation that you should just accept getting injured, but I find it hard to embrace this attitude. When I’m in pain, it's challenging to concentrate because I’m constantly worried about a rupture in my hamstrings or somewhere else.” (P4). Athletes also expressed frustration that despite doing everything possible to prevent injuries (e.g., stretching, using physiotherapy), there remains no assurance that the next injury isn't looming just around the corner. Recognizing that their athletic achievements depend on a functioning body capable for peak performance, this awareness caused a constant sense of anxiety and discomfort in their everyday life.

**Who Am I?** The participants identified strongly with their athletic self since this gave them a sense of competence and believe that they were somehow special. However, the strong identification and perception with their athlete role had also a downside, especially during periods of injuries and prolonged performance slumps. “I believe my weakness was that I identified myself too much as an athlete, and suddenly I didn't have it anymore…so who was I?” (P2). During such periods, athletes were troubled with existential questions about who they were and what their purpose was, thus questioning and doubting their overall value as individuals beyond their achievements in sports.

**I Must Be My Very Best.** The athletes expressed the inner urge to always give their best effort and hold themselves to high standards. They put forward a perfectionistic and self-critical mindset, constantly striving for improvement. This mindset had developed over the years and resulted in feelings of inadequacy and a persistent struggle to meet own expectations: “I constantly focus on performance. Want to be the best version of myself. Didn't do anything that made me happy because I was supposed to improve, improve, improve” (P1). Athletes also experienced self-doubt, especially when they were injured or making a comeback. Despite being international athletes, they worried about their abilities, whether they will be prepared when it counts, or whether they prioritize right things to improve their performance.

**I Blame and Criticize Myself.** Athletes mentioned that they often deal with re-occurring thoughts about not being good enough both as an athlete and as a person in general, and although they know it is just in their head, they struggle to stop these thoughts. P5 explains: “I banged myself on the head many times, like this was stupid! I shouldn't have done this or should have chosen that instead! Why couldn't I just pull myself together?!”. Even though it is common for athletes to engage in negative self-talk and self-criticism, especially when under pressure, this sub-theme reflects their frustration of being so disciplined and goal-orientated on one hand, and not being able to control one's thoughts and emotions, on the other.

### 3.3 Personal resources and strategies

This theme outlines personal resources and strategies athletes found beneficial for nurturing their mental health. Among these strategies are (a) engaging in self-reflection, (b) taking autonomous decisions, (c) planning and prioritization skills, (d) focusing on recovery, (e) knowing one's vulnerabilities, and (f) having love and passion for one's sport and lifestyle.

**I Engage in Self-Reflection.** Athletes emphasized the importance of occasionally taking a step back, acknowledging the progress, and to be proud of personal achievements. In the fast-paced world of elite sport where there is a constant focus on improvement and optimizing, it helps athletes to elaborate on how they are doing and feeling. P4 shared some reflective questions: “What did I learn about myself, how did I develop, and did I act according to my values?”. Athletes underscored the importance of adhering to their values, which provided them with a guiding framework, particularly during times of turmoil and crises. Recognizing the lessons from the past failures and setbacks was regarded as a vital step in cultivating the confidence necessary to tackle upcoming tasks and future challenges.

**I Take Autonomous Decisions.** Athletes commonly expressed a sense of being the architects of their own future, asserting that their decisions played a key role in shaping their career and ultimately determining their success. This includes transitional decisions to change the club or coach, or to prioritize their sport instead of studies or social events in certain periods. “For myself and my career, I had to change clubs because I felt like I didn't belong there anymore. I just think I need to feel connected to the environment I train in” (P2). Athletes stated that they are willing to invest time and energy in relationships that give them energy back but want to dismiss others that were detrimental to their athletic development. Being able to take autonomous decisions was essential for maintaining a sense of control over their lives.

**I Plan and Prioritize My Time.** Athletes found benefits in having a structured plan that helps them to allocate time and resources accordingly. They mentioned that having a weekly overview reduces stress, helping to maintain healthy routines and to include time for rest and recovery. As P4 explained: “I have planned my next weeks and became aware that the calendar is overloaded. If a heavy week is ahead, I need to be mindful… and to incorporate time for mental and physical restitution”. These planning and time-management skills were recognised to be crucial but had to be learned the hard way especially during their dual career struggles in high school. Athletes also stressed the importance of continually improving their ability to remain fully present in the moment and consciously switch between different activities during a day: “I train when I train, I study when I study, and now I do something else” (P5).

**I Focus on Recharging My Batteries.** Athletes noted a heightened awareness of the significance of recovery as they progressed in their careers. This involves not just embracing the latest recovery trends but prioritizing fundamentals like maintaining a consistent sleep schedule and consuming appropriate nutrition. Some athletes found benefits in meditation and acceptance-based approaches as vital strategies to nurture their mental health, especially in periods when things become overwhelming: “I’ve begun practicing meditation, which offers a peaceful space, free from concerns about competition or studies. Here, I can simply unwind and relax.” (P5). Additionally, engaging in social activities that foster a sense of balance, such as going to the movies or enjoying recreational games with friends outside of their team, was emphasized.

**I Know My Vulnerabilities.** Athletes expressed a general understanding that their personality and genetic predisposition plays a role for their mental health. Throughout various stages of their careers, particularly in their late teenage years, all interviewed athletes encountered considerable difficulties regarding their mental health, such as acute stress syndromes, eating disorders, and substance abuse. Nonetheless, with the aid of expert support and interventions encompassing psychoeducation, therapeutic sessions, and, where necessary, medical treatment, these adversities have fostered heightened awareness. The learning of these periods has led to improved literacy and management of their mental health: “I believe, since then, I’ve become much more mindful of my mental health state, and whenever I am feeling down or see warning signs, I react promptly” (P3).

**I Love My Sport and Lifestyle.** Generally, athletes expressed a great love and passion for their sport. Athletes were aware that they are privileged to do what they love for a living, and they expressed that they enjoy many aspects of the elite sport lifestyle that provides structure, meaning, and joy: “I really love what I do. If you have the passion and ambition, then you can get far, even if there are all kinds of challenges and bumps along the way” (P2). For some athletes, the passion for their sport served as a valuable resource during times of personal turmoil and difficult periods when they were struggling with mental health issues: “I am convinced that my passion for my sport has saved my life in a way” (P3).

### 3.4 Supportive and caring environment

Holistic support and relationships with key people in the athletes' environment were perceived to be nurturing their mental health. Specifically, these positive environmental factors include (a) positive relationship with coaches, (b) culture of openness, (c) supportive teammates, (d) expert support network, (e) dual career support, and (f) unconditional love from others.

**My Coach Understands Me.** Athletes emphasized the significance of empathetic understanding as a crucial quality of a supportive coach for their mental health. “My coach is just an incredibly pleasant person. I think he is the best coach I have ever had, not only because of his sporting expertise but because of his human qualities” (P2). This entails coaches being able to empathize with the athletes' experiences and demonstrating willingness to engage in discussions beyond training and tactics. “I can talk with my coach about everything” (P3). Establishing enduring and close relationships with their athletes, coaches were especially valuable during challenging times and personal crises, showing a genuine interest in the person beyond their performance.

**We Can Openly Discuss Our Concerns.** Athletes recognized the importance of seeking assistance, understanding that without reaching out or sharing their concerns, they cannot expect support. Developing this willingness to seek help requires courage and represents a skill athletes needed to cultivate throughout their careers. As P1 reflected: “It just helps so much to express and share my worries and issues. If I didn't do that, I am pretty sure it would have gone really wrong, because we [athletes] tend to believe we can manage everything by ourselves.” Athletes valued the opportunity to openly discuss their daily struggles within their team, even when they didn't require immediate solutions. Athletes emphasized the importance of recognizing the delicate balance between expressing curiosity and inquiring, while also respecting the possibility that teammates may not wish to share or discuss their personal matters and mental health.

**We Have Each Other's Back.** Both individual and team sports athletes emphasized the importance of having strong bonds and supportive relationships with their teammates. Athletes simply spend so much time together and depend on each other in terms of performing at a higher level. Teammates can also offer support during times of defeat and difficult situations when negative emotions prevail: “My teammate has always had my back, and we share a strong friendship. Our communication is respectful and effective, and we are good to handling defeats together.” (P1). Feeling of belonging to the team, with mutual support and respect, were regarded as central factors for athletes' daily thriving within their training environment.

**I Can Count on Experts’ Support.** Athletes mentioned a range of professionals who provide help for their physical and mental health. These include mental coaches, psychologists, doctors, physiotherapists, and nutrition specialists. Athletes acknowledged that although these experts are part of their network and potentially available, it is their proactive approach in seeking help that makes a difference. A well-coordinated effort from the specialists surrounding the athlete is most beneficial, and P4 emphasized the importance of having quick access to assistance when needed: “I inquired about seeing a doctor, and the very next day, I underwent a scan. This efficient process provided a swift path to a solution, sparing me from prolonged speculation and having negative thoughts.”

**I Get Help for My Dual Career.** The interviewed athletes felt well supported within the Danish dual career system in terms of flexibility and assistance from qualified support staff, highlighting particularly the dual career benefits related to higher education. The dual career provides them with a sense of balance, personal development, meaning, and autonomy (e.g., choosing the study subject), even though there may be periods of increased effort throughout the year. Especially during these stressful periods, receiving assistance with rescheduling exams, receiving extra tutoring, or planning the semester were immensely valuable. P3 shared: “I had incredibly helpful dual career support providers who guided me in setting priorities and ensuring my overall well-being. Without this specialized service, it would have been impossible.”

**I Have People Who Love Me.** Family and parental support played a pivotal role in the lives of all athletes throughout their careers. Parents consistently stood by their children, offering support especially during challenging times. Athletes mentioned that they occasionally discuss personal issues with their parents but typically provide a more general overview to prevent their parents from becoming overly concerned. According to the athletes, the most important aspect of parental support was simply having someone who would listen and understand. In addition to parents, athletes also pointed to their partners and friends outside of the sporting world as sources of unconditional love, irrespective of their athletic performance. In these relationships, athletes feel they can be themselves without the need to wear a mask. “I live together with my boyfriend who truly cares about me as a person. Then also my brothers and family, and friends outside sport. I am fortunate to have many people I can rely on” (P6).

## 4 Discussion

The aim of the current study was to conduct an in-depth exploration of the dynamic interplay of factors perceived by Danish elite athletes as influencing their mental health. Athletes articulated a myriad of factors, stemming from both external and internal sources, which they identified as either detrimental (risk) or beneficial (protective) to their mental health. Through the presentation of the thematic map (Figure 1), we aimed to streamline the complexity of the various factors while maintaining the dynamic interplay among them, illustrated by the arrows connecting the overarching themes. Following, we discuss the thematic map with a focus on the factors' interplay, moving from the environmental to the personal factors that athletes identified as impacting their mental health.

### 4.1 Nurturing environments can compensate for the relentless elite sport context

Different aspects of the elite sport context were perceived as a threat to mental health, including some of the risk factors that were identified in the scoping review by Kuettel and Larsen (3). For example, sport-specific stressors (e.g., pressure, deselection, weight control (23); resonate well with the subthemes identified in this study around *Perform and deliver!* and *Push through it!* Many of these elite sport stressors mentioned have previously been categorized by Arnold and Fletcher (24) as logistical and environmental issues (e.g., selection, travel), team issues (e.g., teammates behaviour, cultural norms), and leadership and personnel issues (e.g., coach behaviour, external expectations). Another risk factor identified in Kuettel and Larsen's review (3) was lack of social support from teammates and coaches. Our findings highlighted the dual role that coaches and teammates play in relation to athletes' mental health, supporting the conclusions drawn by Pankow et al. (25), who identified supportive and punitive coach behaviours as protective and risk factors, respectively.

The athletes interacted with different coaches throughout their careers. In coach-athlete relationships where performance was emphasized over mental health, athletes' mental health was affected negatively, fostering feelings of not being taken seriously which could result in mistrust, conflicts, and in some cases even bullying. In other examples, athletes highlighted coaches as central contributors to their sporting development and mental health, emphasizing the significance of elements like closeness (built on trust and respect) and co-orientation (rooted in mutual understanding) in the relationship (26). These aspects, along with a caring approach (27), were seen as essential by the athletes to nurture their mental health, especially during times of personal struggles and performance slumps. Coaches, often regarded as cultural leaders (28), wield considerable influence over team dynamics and can create a psychologically safe environment where athletes feel at ease to show their authentic self and where mental health talks are normalized (29).

Athletes participating in this study generally felt comfortable around their teammates and emphasized the positive culture in which they could openly discuss and challenge each other. A recent report (30) found that less than 1% of the Danish elite athletes experienced boundary-crossing behaviour from teammates or coaches on a weekly basis. Nevertheless, the athletes also mentioned periods during their athletic career when they were integrated into subcultures marked by the prevalence of stigma around mental illness and the imposition of taboos (10). This added to their mental health difficulties, particularly during their late teens when they faced multiple stressors related to their dual careers (5), a period where the risk of developing a mental disorder is heightened (31). A quantitative study involving Danish soccer players found an increase in stressors and heightened symptoms of depression, alongside a decrease in mental health levels at age 18, supporting these findings (32).

Athletes emphasised the importance of having a dual career that gave them a sense of balance and contributed to their general development as human beings. Help for their dual career and collaboration of experts ensured that the burden of coordinating efforts didn't solely rest on the shoulders of the athlete. Case studies conducted in Danish dual career environments have highlighted the crucial role of a well-functioning dual career support team that can help athletes with planning and prioritizing, and to have focus on mental health (12). Moreover, the provision of general social support and the feeling of being loved and accepted by significant others (e.g., parents, partners, friends) were perceived as contributing to the overall mental health of the athletes. Altogether, while the athletes occasionally found the elite sports environment to be relentless and a threat to their mental health, they also observed that a supportive and functional environment acted as a nurturing and protective factor.

### 4.2 Personal resources and strategies can nurture mental health

The cultural theory of learning (33) highlights how learning cultures shape our habitual actions within specific contexts and situations. Regarding elite sports, this notion suggests that athletes' self-perceptions, actions, and approaches are heavily influenced by the prevailing norms and values of the elite sport context (34). Participants' personal reactions to the relentless elite sports context were expressed as a constant struggle with injuries, worries about the future, identity issues, perfectionistic concerns, self-blame, and criticism. Ineffective coping (e.g., difficulties in expressing emotions and reduced help-seeking (23); or perfectionistic concerns (4) were previously identified risk factors that resemble the subthemes about self-blame and criticism, as well as athletes' high personal standards and unrealistic expectations for constant improvement.

To counterbalance these negative factors, athletes mentioned to have developed various resources and strategies over the course of their career. Among the nurturing factors at the internal level was the athletes' awareness of recovery that included physiological (e.g., sleep habits, massage, sauna,), social (e.g., cinema with friends) or psychological (e.g., mindfulness) practices. However, their awareness of recovery had developed over time, supported by the collaboration with sport psychologists. Further, dual career competencies (35) such as time management, planning and prioritizing, and emotional awareness were highlighted as factors supporting one's career excellence and “winning in the long-run” (6).

Knowing one's vulnerabilities was seen as a key factor to be mindful of one's current mental health state. This awareness was heightened based on the periods of developmental crises and the corresponding occurrence of mental health issues, which could be described as a mismatch between athletes' resources (the positive/nurturing factors) and demands (the negative/threatening factors). In retrospect, the learnings of these periods were crucial for growth following adversity (36) and helped the athletes to become the persons they are today. Therefore, coping with career transitions and developmental crises can be interpreted as inherent parts of athletes' pursuit of career excellence (6). During the interviews, the athletes came to realize that their overall mental health literacy (9) was lower at the time of crises compared to their current level. This discrepancy also influenced their willingness to openly share their struggles and actively seek assistance (8). On top, when injuries made athletes to question their future and identity, combined with inappropriate coping strategies and negative self-talk, it led to mental health issues that needed attention and in some cases treatment. Insofar, it was not a single risk factor that made athletes develop mental health issues in certain career phases, but rather the complex combination of several negative personal and environmental factors that put athletes at risk.

### 4.3 Elite sport lifestyle as a double-edged sword

Even though we have attempted to group mental health factors in Figure 1 into positive/nurturing (green) and negative/threating ones (red), we recognize that the categorization does not consider the dual nature of some factors. Previously, we have outlined the adverse aspects of the high-pressure elite sport environment and the associated negative repercussions on athletes' mental health at the personal level. Despite these struggles, the athletes universally conveyed a sense of profound gratitude for being able to pursue their greatest passion as a career and to fully embrace the elite sport lifestyle. Gratitude has been identified as one of the key elements of mental health (37). Moreover, athletes perceived their elite sport lifestyle as offering significant autonomy, such as making their own decisions and setting personal goals. They also expressed a strong sense of competence and uniqueness, deriving from their exceptional skills and achievements in sport, which further contributed to their athletic identity that was seen as a resource for their mental health (6, 7).

Other positive factors of the elite sport lifestyle included the importance of meaningful relationships both within and outside the sports context, as emphasized by all athletes. Altogether, athletes expressed that autonomy, competence, and relatedness as the three basic human needs (38) appeared to be essential for facilitating their functioning, growth, and mental health. Basic psychological needs satisfaction was also found to be a strong predictor of Swiss athletes' mental health (39). Remarkably, many of the nurturing factors (e.g., trusting personal relationships, functional coping strategies, feeling of autonomy, and value-based decisions) mentioned by the athletes are integrated parts of the mental health definition by Kuettel and Larsen (3) presented earlier.

### 4.4 The benefits and crux of self-reflection

Athletes mentioned that they have used self-reflection throughout their career, starting with reflection about their strengths and weaknesses in terms of sporting development and performance, and later by reflecting over their values and life more in general (40). Engaging in structured self-reflection to build up confidence and to gain personal insights has been highlighted to be an important psychological skill to reach the elite level (41) and a predictor of enhanced resilience in athletes (42). Insofar, self-reflection emerged as a key element intertwined with various positive personal resources and strategies highlighted in our study. These include recognizing one's vulnerabilities, understanding when and how to prioritize recovery, and making informed choices and decisions concerning the dual-career lifestyle (5, 6).

On the downside, the constant self-evaluation of performance outcomes and high personal standards in the athletic, academic, and private domain led to self-doubt, self-blame, and a generally harsh tone towards oneself among the interviewed Danish elite athletes. While some athletes described rumination as a strategy to cope with stressors and adversity, the tendency to repeatedly focus on negative events and thoughts was dysfunctional and consequently led to greater distress in the long run (43). Corroborating these findings, Tahtinen and colleagues (44) have shown that British athletes with higher levels of rumination had a higher risk of experiencing clinical levels of depressive symptoms.

In summary, the impact of events, situations, and environments on athletes' mental health is neither inherently positive nor negative; instead, their influence depends on how these factors are perceived and managed. For instance, factors like coaching, team dynamics, injuries, or stressful periods can contribute to both positive and negative mental health outcomes, contingent upon the presence of a supportive environment and the athlete's coping resources and strategies. The Latin phrase *Quod me nutrit, me destruit* (“that which nourishes me destroys me”) might serve as a fitting analogy for the complex interplay of risk and protective factors in elite sports as they impact athlete mental health.

### 4.5 Limitations

Several limitations of this study should be noted. Firstly, all the participating Danish athletes had access to sport psychology services, which could influence their responses. Yet, considering naturalistic generalizability (16) and based on our experiences in international elite sport contexts, we believe the findings will resonate with elite athletes outside of Denmark and regardless of access to sport psychology support. Secondly, the thematic analysis, which involved extracting factors from individual interviews and presenting the findings in themes, cannot fully capture the dynamic nature of these factors throughout the athletes' career development. Thus, future research should aim to investigate this dynamism by examining how these factors evolve across different career stages or transitions. Thirdly, gender differences regarding factors were not accounted for in the present study. Research has revealed gender differences in help-seeking behavior (8), and female athletes have in general reported lower mental health levels than males (4). These differences were not apparent in our data; hence, the factors are presented as gender neutral in the thematic map. Finally, the thematic map is presented in an orthogonal format, which does not fully capture the duality and complexity of the relationships between factors. While no model is perfect, it serves as an attempt to simplify the complex phenomena of the many factors affecting athletes' mental health.

### 4.6 Practical implications

Consistent with the recommendations of the International Society of Sport Psychology (7), the present study highlights the need for creating psychologically safe environments (10) that can nurture athletes' mental health through supporting athletes' autonomy and facilitating team cultures where sharing mental health struggles is normalized. The Team Danmark applied model of mental health (14) provides a helpful lens for practitioners to work with prevention within the holistic ecological approach. Moreover, athletes emphasized the value of mental health education, yet expressing regret that they acquired this knowledge (too) late in their careers. This underscores the significance of implementing mental health literacy programs (9) early in the talent development phase. Additionally, coaches and sport psychology consultants are encouraged to assist athletes in cultivating the self-reflection skills both within their sport and personal lives (42). This can be achieved through facilitating constructive self-evaluations and emphasizing the implementation of effective recovery strategies and by developing an individualized wellbeing development plan (45). With youth athletes who have high self-expectations and a single-minded focus on success—often at the expense of their mental health—it is valuable to discuss the dual nature of these factors and the importance of finding balance. The participating athletes noted that this sense of balance was something they only gained with greater experience and maturity. Their experiences and insights could therefore serve a mentoring role, helping younger athletes become more aware of the importance of mental health in pursuing a sustainable career. Finally, when supporting athletes engaged in a dual career, it is important to be mindful of critical career periods and to provide extra support during the late teenage years when transitions in several life spheres happen simultaneously, increasing the risk of developing a mental health disorder.

## 5 Conclusions

Given the increased interest in the mental health of elite sport performers and a dominance of quantitative prevalence studies, the present qualitative study provides a timely investigation into elite athletes' perception of factors that impact their mental health. We present a thematic map consisting of four overarching themes to illustrate the complexity and interplay of these factors. To counterbalance a relentless elite sport context and the personal struggles connected with an elite lifestyle, the interviewed athletes emphasized the importance of personal resources and strategies alongside a supportive and caring environment. However, we also discussed the dual nature of certain factors and their impact on athletes' mental health. The current study fills an important gap in the literature by examining not only risk factors for athletes' mental health, but also factors that contribute to their thriving and pursuit of career excellence from a holistic developmental and ecological perspective.

## Data Availability

The raw data supporting the conclusions of this article will be made available by the authors, without undue reservation.

## References

[1] Lundqvist C Andersson G. ‘Let’s talk about mental health and mental disorders in elite sports: a narrative review of theoretical perspectives’. Front Psychol. (2021) 12:700829. 10.3389/fpsyg.2021.700829 34267715 PMC8275956

[2] Reardon CL Hainline B Aron CM Baron D Baum AL Bindra A Mental health in elite athletes: international Olympic committee consensus statement (2019). Br J Sports Med. (2019) 53(11):667–99. 10.1136/bjsports-2019-100715 31097450

[3] Kuettel A Larsen CH. Risk and protective factors for mental health in elite athletes: a scoping review. Int Rev Sport Exerc Psychol. (2020) 13(1):231–65. 10.1080/1750984X.2019.1689574

[4] Rice SM Purcell R De Silva S Mawren D McGorry PD Parker AG. The mental health of elite athletes: a narrative systematic review. Sports Med. (2016) 46:1333–53. 10.1007/s40279-016-0492-2 26896951 PMC4996886

[5] Kegelaers J Wylleman P Defruyt S Praet L Stambulova N Torregrossa M The mental health of student-athletes: a systematic scoping review. Int Rev Sport Exerc Psychol. (2022):1–34. 10.1080/1750984X.2022.2095657

[6] Stambulova NB Ryba TV Henriksen K. Career development and transitions of athletes: the international society of sport psychology position stand revisited. Int J Sport Exerc Psychol. (2021) 19(4):524–50. 10.1080/1612197X.2020.1737836

[7] Henriksen K Schinke R Moesch K McCann S Parham WD Larsen CH Consensus statement on improving the mental health of high performance athletes. Int J Sport Exerc Psychol. (2020) 18(5):553–60. 10.1080/1612197X.2019.1570473

[8] Cosh S McNeil D Jeffreys A Clark L Tully P. Athlete mental health help-seeking: a systematic review and meta-analysis of rates, barriers and facilitators. Psychol Sport Exerc. (2024) 102586. 10.1016/j.psychsport.2023.102586 38128709

[9] Kutcher S Wie Y Coniglio C. Mental health literacy: past, present, and future. Can J Psychiatr. (2016) 61(3):154–8. 10.1177/0706743715616609 PMC4813415 27254090

[10] Walton CC Purcell R Pilkington V Hall K Kenttä G Vella S Psychological safety for mental health in elite sport: a theoretically informed model. Sports Med. (2023) 54(3):1–8. 10.1007/s40279-023-01912-2 37737542 PMC10978613

[11] Danish Elite Sport Act. Retsinformation (2021). Available online at: https://www.retsinformation.dk/eli/lta/2021/785 (accessed June 18, 2024).

[12] Henriksen K Storm LK Küttel A Linnér L Stambulova N. A holistic ecological approach to sport and study: the case of an athlete friendly university in Denmark. Psychol Sport Exerc. (2020) 47:101637. 10.1016/j.psychsport.2019.101637

[13] Kuettel A Christensen MK Zysko J Hansen J. A cross-cultural comparison of dual career environments for elite athletes in Switzerland, Denmark, and Poland. Int J Sport Exerc Psychol. (2020) 18(4):454–71. 10.1080/1612197X.2018.1553889

[14] Henriksen K Diment G Kuettel A. The team Denmark applied model of athlete mental health. Int J Sport Exerc Psychol. (2023):1–17. 10.1080/1612197X.2023.2281525

[15] Kuettel A Pedersen AK Larsen CH. To flourish or languish, that is the question: exploring the mental health profiles of Danish elite athletes. Psychol Sport Exerc. (2021) 52:101837. 10.1016/j.psychsport.2020.101837

[16] Smith B Sparkes AC. Routledge Handbook of Qualitative Research in Sport and Exercise. London: Routledge (2016).

[17] Patton MQ. Qualitative Research and Evaluation Methods. 4th ed. Thousand Oaks, CA: Sage (2015).

[18] Swann C Moran A Piggott D. Defining elite athletes: issues in the study of expert performance in sport psychology. Psychol Sport Exerc. (2015) 16(1):3–14. 10.1016/j.psychsport.2014.07.004

[19] Braun V Clarke V. Thematic Analysis: A Practical Guide. London: Sage (2022).

[20] Schubring A Mayer J Thiel A. Drawing careers: the value of a biographical mapping method in qualitative health research. Int J Qual Methods. (2019) 18:1–12. 10.1177/1609406918809303

[21] Kvale S. Doing Interviews. Thousand Oaks, CA: Sage (2007).

[22] Smith B McGannon KR. Developing rigor in qualitative research: problems and opportunities within sport and exercise psychology. Int Rev Sport Exerc Psychol. (2018) 11(1):101–21. 10.1080/1750984X.2017.1317357

[23] Gulliver A Griffiths KM Christensen H. Barriers and facilitators to mental health help-seeking for young elite athletes: a qualitative study. BMC Psychiatry. (2012) 12:157. 10.1186/1471-244X-12-157 23009161 PMC3514142

[24] Arnold R Fletcher D. A research synthesis and taxonomic classification of the organizational stressors encountered by sport performers. J Sport Exerc Psychol. (2012) 34(3):397–429. 10.1123/jsep.34.3.397 22691400

[25] Pankow K Sutcliffe JT Conyers D Robinson LD Schweickle MJ Liddelow C Mental health risk and protective factors in Australian cricket. Int J Sport Exerc Psychol. (2024):1–22. 10.1080/1612197X.2024.2310103

[26] Jowett S. Coaching effectiveness: the coach-athlete relationship at its heart. Curr Opin Psychol. (2017) 16:154–8. 10.1016/j.copsyc.2017.05.006 28813341

[27] Dohsten J Barker-Ruchti N Lindgren E-C. Caring as sustainable coaching in elite athletics: benefits and challenges. Sports Coach Rev. (2020) 9(1):48–70. 10.1080/21640629.2018.1558896

[28] Storm LK Svendsen AM Stambulova N Barker D Ronkainen N Bjørndal CT Cultural leadership in physical education and youth sport: consensus from a nordic think tank. Scand J Sport Exerc Psychol. (2024) 6:1–9. 10.7146/sjsep.v6i.141284

[29] Jowett S Do Nascimento-Júnior JRA Zhao C Gosai J. Creating the conditions for psychological safety and its impact on quality coach-athlete relationships. Psychol Sport Exerc. (2023) 65:102363. 10.1016/j.psychsport.2022.102363 37665836

[30] TD Trivselsundersøgelse. Team Denmark well-being report. IDAN (2023). Available online at: https://www.idan.dk/udgivelser/team-danmark-atletundersoegelse-2023/ (accessed June 18, 2024).

[31] Åkesdotter C Kenttä G Eloranta S Franck J. The prevalence of mental health problems in elite athletes. J Sci Med Sport. (2020) 23(4):329–35. 10.1016/j.jsams.2019.10.022 31806359

[32] Kuettel A Durand-Bush N Larsen CH. Mental health profiles of Danish youth soccer players: the influence of gender and career development. J Clin Sport Psychol. (2021) 16(3):276–93. 10.1123/jcsp.2021-0035

[33] Hodkinson P. Learning as cultural and relational: moving past some troubling dualisms. Camb J Educ. (2005) 35(1):107–19. 10.1080/0305764042000332524

[34] Barker-Ruchti N. Athlete Learning in Elite Sport. London: Routledge (2019).

[35] De Brandt K Wylleman P Torregrossa M Schipper-Van Veldhoven N Minelli D Defruyt S Exploring the factor structure of the dual career competency questionnaire for athletes in European pupil-and student-athletes. Int J Sport Exerc Psychol. (2018):1–18. 10.1080/1612197X.2018.1511619

[36] Howells K Wadey R Roy-Davis K Evans L. A systematic review of interventions to promote growth following adversity. Psychol Sport Exerc. (2020) 48:101671. 10.1016/j.psychsport.2020.101671

[37] Van Agteren J Iasiello M Lo L Bartholomaeus J Kopsaftis Z Carey M A systematic review and meta-analysis of psychological interventions to improve mental wellbeing. Nature Human Behaviour. (2021) 5:1–22. 10.1038/s41562-021-01093-w 33875837

[38] Ryan RM Deci EL. Self-determination theory and the facilitation of intrinsic motivation, social development, and well-being. Am Psychol. (2000) 55(1):68. 10.1037/0003-066X.55.1.68 11392867

[39] Röthlin P Horvath S Ackeret N Peter C Birrer D. The mental health of Swiss elite athletes. Swiss Psychol Open. (2023) 3(1):1–17. 10.5334/spo.49

[40] Henriksen K. The values compass: helping athletes act in accordance with their values through functional analysis. J Sport Psychol Action. (2019) 10(4):199–207. 10.1080/21520704.2018.1549637

[41] MacNamara A. Psychological characteristics of developing excellence. In: Collins D Abbott A Richards H, editors. Performance Psychology for Physical Challenge. Churchill Livingstone: Elsevier (2011). p. 47–62.

[42] Cowden RG Meyer-Weitz A. Self-reflection and self-insight predict resilience and stress in competitive tennis. Soc Behav Pers. (2016) 44(7):1133–50. 10.2224/sbp.2016.44.7.1133

[43] McLoughlin E Arnold R Moore LJ Slavich GM Fletcher D. A qualitative exploration of how lifetime stressor exposure influences sport performers’ health, well-being, and performance. Anxiety Stress Coping. (2023) 37(2):1–18. 10.1080/10615806.2023.2246023 37665577 PMC11216060

[44] Tahtinen R McDougall M Feddersen N Tikkanen O Morris R Ronkainen NJ. Me, myself, and my thoughts: the influence of brooding and reflective rumination on depressive symptoms in athletes in the United Kingdom. J Clin Sport Psychol. (2019) 14(3):285–304. 10.1123/jcsp.2019-0039

[45] Hoare E Couston N Burdon L Vella C Hall K. Feasibility and acceptability of a multi-component mental wellbeing program for young high-performance athletes in Australian Rules Football. Front Sports Act Living. (2024) 6:1470726. 10.3389/fspor.2024.1470726 39640506 PMC11617179

